# NADPH Oxidase/ROS-Dependent VCAM-1 Induction on TNF-α-Challenged Human Cardiac Fibroblasts Enhances Monocyte Adhesion

**DOI:** 10.3389/fphar.2015.00310

**Published:** 2016-01-28

**Authors:** Chih-Chung Lin, Chien-Chung Yang, Chen-Yu Wang, Hui-Ching Tseng, Chih-Shuo Pan, Li-Der Hsiao, Chuen-Mao Yang

**Affiliations:** ^1^Department of Anesthetics, Chang Gung Memorial Hospital at Linkou, and College of Medicine, Chang Gung UniversityTao-Yuan, Taiwan; ^2^Department of Physiology and Pharmacology and Health Aging Research Center, College of Medicine, Chang Gung UniversityTao-Yuan, Taiwan; ^3^Department of Traditional Chinese Medicine, Chang Gung Memorial Hospital at Lin-KouTao-Yuan, Taiwan; ^4^Research Center for Industry of Human Ecology and Graduate Institute of Health Industry Technology, Chang Gung University of Science and TechnologyTao-Yuan, Taiwan

**Keywords:** TNF-α, VCAM-1, NADPH oxidase, ROS, transcription factor, cardiac fibroblasts

## Abstract

The inflammation-dependent adhesion molecule expressions are characterized in cardiovascular diseases and myocardial tissue infiltrations. Several pro-inflammatory cytokines are elevated in the acute myocardial injury and infarction. Tumor necrosis factor-α (TNF-α), a pro-inflammatory cytokine, is raised in the injury tissues and inflammatory regions and involved in the pathogenesis of cardiac injury, inflammation, and apoptosis. In fibroblasts, TNF-α-triggered expression of vascular cell adhesion molecule (VCAM)-1 aggravated the heart inflammation. However, the mechanisms underlying TNF-α-mediated VCAM-1 expression in cardiac fibroblasts remain unclear. Here, the primary cultured human cardiac fibroblasts (HCFs) were used to investigate the effects of TNF-α on VCAM-1 expression. The molecular evidence, including protein, mRNA, and promoter analyses, indicated that TNF-α-induced VCAM-1 gene expression is mediated through the TNFR-dependent manner. Activation of TNF-α/TNFR system triggered PKCα-dependent NADPH oxidase (Nox)/reactive oxygen species (ROS) signal linking to MAPK cascades, and then led to activation of the transcription factor, AP-1. Moreover, the results of mRNA and promoter assay demonstrated that c-Jun/AP-1 phosphorylated by TNF-α turns on VCAM-1 gene expression. Subsequently, up-regulated VCAM-1 on the cell surface of TNF-α-challenged HCFs increased the number of monocytes adhering to these cells. These results indicated that in HCFs, activation of AP-1 by PKCα-dependent Nox/ROS/MAPKs cascades is required for TNF-α-induced VCAM-1 expression. To clarify the mechanisms of TNF-α-induced VCAM-1 expression in HCFs may provide therapeutic strategies for heart injury and inflammatory diseases.

## Introduction

Cardiovascular diseases are characterized by overexpression of inflammatory genes and infiltration of the inflammatory cells via adhesion molecule induction such as VCAM-1 (Bedard and Krause, 2007; Cheng et al., 2009; Cook-Mills et al., 2011). Previous report indicates that the elevated levels of adhesion molecules (e.g., VCAM-1) have been detected in the serum of non-ischemic heart failure patients and the inflammatory infiltrations in the myocardial tissue (Cronstein, 1994). In polymorphonuclear cells (PMNs), induction of adhesion molecules can mediate the tight cellular adhesiveness and thus facilitate PMNs migration across the vascular endothelial barrier (Dustin et al., 1988; Frangogiannis et al., 2002). The cardiac fibroblasts exhibit versatile ability through the potential to activate a series of genes that initiate and amplify inflammation in heart diseases. Additionally, up-regulation of VCAM-1 and tumor necrosis factor-α (TNF-α) can be detected in the cardiac vascular endothelium of chronic heart failure patients (Cronstein, 1994; Savic-Radojevic et al., 2013). Therefore, up-regulation of VCAM-1 expression on cytokine-triggered heart diseases may be the key factor responsible for the targeted leukocyte transmigration into extra-vascular space of inflammation (Dustin et al., 1988; Frangogiannis et al., 2002).

Heart diseases are associated with acute myocardial injury initiating a sequence of events that often lead to cardiac hypertrophy, chamber dilation, interstitial fibrosis, and congestive heart failure (Communal and Braunwald, 2005). Several pro-inflammatory cytokines are elevated in patients and animal models of acute myocardial injury and infarction, which may cause phenotypic and functional changes in the constituent cell types of the heart (Javaid et al., 2003; Hsieh et al., 2012; Kim et al., 2012), including generation of various mediators by cardiac fibroblast (Klein et al., 1998; Kono et al., 2003; Kim et al., 2013). Therefore, chronically raised pro-inflammatory cytokines, including TNF-α, IL-6, IL-1, and IL-18, could affect the cardiac structure and function during heart injury and infarctions. Such as TNF-α and IL-6, these pro-inflammatory cytokines play key roles in the pathological process dependent on their concentrations in primary insults (Koudssi et al., 1998; Hedayat et al., 2010). Among these, TNF-α may be a prominent cytokine in the pathogenesis of cardiac injury, inflammation, and apoptosis (LeBel et al., 1992; Kyriakis and Avruch, 2001). TNF-α triggers up-regulation of many genes (including adhesion molecules) to initiate inflammatory responses (Lee et al., 2006). Recent study also showed that inhibition of TNF-α-mediated pathways reduces myocardial ischemia-reperfusion injury (Kyriakis and Avruch, 2001). These results suggest that TNF-α exerts potential mediators in heart inflammatory diseases.

Reactive oxygen species (ROS) are generated by several enzymatic reactions and chemical processes or directly inhaled, including $O_{2}^{\cdot -}$, OH^⋅^, and hydrogen peroxide (H_2_O_2_). Low levels of these oxidative-contained molecules are required for maintaining normal physiological functions and protecting against invading microorganisms (Lee et al., 2008). Among these, various approaches indicate that NADPH oxidase (Nox) complex is considered to be a major source of ROS generation (Rahman et al., 2006; Lin et al., 2012; Lee and Yang, 2013). It has been shown that overproduction of ROS acts as a risk factor leading to impairing cellular functions and enhancing inflammatory responses (Kolluru et al., 2012; Lee and Yang, 2012). In the cardiovascular system, low levels of ROS contribute to maintenance of cellular redox homeostasis. Under pathological states, excessive ROS production by several pro-inflammatory mediators like cytokines can induce expression of various inflammatory genes during heart disorders (Bhagat and Vallance, 1997; Libby, 2002; Lin et al., 2015). Accumulating evidences have indicated that ROS generation acts as a deleterious signal that leads to damage of heart cells and heart inflammatory injuries/diseases. ROS are also known to be implicated in the pathogenesis and deterioration of various cardiac disorders, such as myocardial infarction (MI) and heart failure (Looi et al., 2008; Hori and Nishida, 2009; Lin et al., 2015). Therefore, increasing evidence has shown that Nox-derived ROS production may contribute to regulation of several inflammatory mediators in heart diseases (Lambeth, 2007; Yang et al., 2013a). However, the pathways of VCAM-1 expression via TNF-α-initiated ROS generation remain to be defined in human cardiac fibroblasts (HCFs).

TNF-α induces expression of several inflammatory genes by activation of TNF receptor (TNFR)-mediated diverse signaling molecules, including PKCs, ROS, MAPKs, and transcription factors (Palmer et al., 1995; Lin et al., 2012). Previous studies suggest that the MAPK pathway is involved in several pathophysiological processes of heart diseases (Rajesh et al., 2003; Palomer et al., 2013). In addition, p42/p44 MAPK, p38 MAPK, and JNK1/2, the three well characterized MAPK subfamilies, are the targets of pharmacological and genetic manipulations to explore their roles in cardiac development, function, and diseases (Ravingerova et al., 2003). Moreover, activation of MAPKs leading to expression of inflammatory genes by ROS has been shown in various cell types (Reinehr et al., 2007). The previous studies have also shown that MAPK activation is involved in the up-regulation of VCAM-1 in various cell types (Reinehr et al., 2007; Rose et al., 2010; Lee et al., 2012). Recent study demonstrated the involvement of Nox-dependent ROS generation in TNF-α-induced MMP-9 expression in rat heart-derived H9c2 cells (Kim et al., 2012; Yang et al., 2013a). The transcription factor activator protein 1 (AP-1) is critical for inflammatory processes induced by various stimuli including cytokines (Lee et al., 2006; Saito et al., 2012). Moreover, the expression of VCAM-1 and other genes appears to be up-regulated by ROS and AP-1 in various cell types (Siwik et al., 2000; Reinehr et al., 2007; Savic-Radojevic et al., 2013). VCAM-1 promoter also contains AP-1 binding sites which are regulated by TNF-α through MAPKs-mediated pathways in several cell types (Szekanecz et al., 1996; Siwik et al., 2000). Therefore, we examined whether these signaling components are involved in the TNF-α-induced VCAM-1 expression in HCFs.

In the study, to investigate the molecular mechanisms of TNF-α-mediated responses in HCFs, we found that Nox/ROS-mediated pathways were involved in VCAM-1 induction. These results demonstrated that TNF-α induces TNFR1/PKCα-dependent Nox/ROS/MAPKs cascade in HCFs. Through this pathway, TNF-α promoted the activation of c-Jun/AP-1 to increase VCAM-1 transcription and resulting in the VCAM-1 protein expression in HCFs. Moreover, we found that the increased VCAM-1 expression contributes with monocytes adhesion to TNF-α-challenged HCFs.

## Materials and Methods

### Materials

DMEM/F-12 medium, fetal bovine serum (FBS), TRIzol reagent and PLUS-Lipofectamine were from Invitrogen (Carsbad, CA, USA). Hybond C membrane and enhanced chemiluminescence (ECL) detection system were from GE Healthcare Biosciences (Buckinghamshire, UK). PhosphoPlus PKCα (#9375), p42/p44 MAPK (#9101), p38 MAPK (#9211), JNK1/2 (#4668), and c-Jun (#2361) antibody kits were from Cell Signaling (Danver, MA, USA). VCAM-1 (sc-8304), PKCα (sc-208), Nox2 (sc-20782), Nox4 (sc-30141), p42 (sc-154), p38α (sc-535), JNK1 (sc-474), and c-Jun (sc-1694) antibody were from Santa Cruz (Santa Cruz, CA, USA). Ro31-8220, Gö6976, Edaravone, dipheneyleneiodonium chloride (DPI), U0126, SB202190, SP600125, and tanshinone IIA (TSIIA) were from Biomol (Plymouth Meeting, PA, USA). Apocynin (APO) was from Cayman (Ann Arbor, MI, USA). GAPDH antibody was from Biogenesis (Bournemouth, UK). Bicinchoninic acid (BCA) protein assay kit was from Pierce (Rockford, IL, USA). SDS-PAGE reagents were from MDBio Inc (Taipei, Taiwan). Enzymes and other chemicals were from Sigma (St. Louis, MO, USA).

### HCFs Culture and Treatment

Human cardiac fibroblasts isolated from human fetal heart were obtained from ScienCell Research Laboratories (Carlsbad, CA, USA) and grown in DMEM/F-12 containing 10% FBS, 2 mM glutamine and antibiotics (100 U/ml penicillin G, 100 μg/ml streptomycin, and 250 ng/ml fungizone) at 37°C. Reaching confluence, cells were treated with 0.05% trypsin/0.53 mM EDTA and plated onto 12-well culture plates and made quiescent at confluence by incubation in serum-free DMEM/F-12 for 24 h, and then incubated with TNF-α at 37°C for the indicated time intervals. When the inhibitors were used, cells were pretreated with the inhibitors for 1 h before exposure to TNF-α. Treatment of HCFs with DMSO, pharmacological inhibitors, or TNF-α alone had no significant effect on cell viability determined by an XTT assay (data not shown). Experiments were performed using cells from passages 3 to 6.

### Preparation of Cell Extracts and Western Blot Analysis

After treatment with TNF-α, HCFs were washed with ice-cold PBS, scraped, and collected by centrifugation at 45,000×*g* for 1 h at 4°C to yield the whole cell extract, as previously described (TorreAmione et al., 1996). Samples were denatured and separated by 10% SDS-PAGE. The transferred nitrocellulose membranes were blocked with 1% BSA in TTBS (50 mM Tris-HCl, pH 7.4; 150 mM NaCl; 0.1% Tween 20) for 30 min and then probed with a respective primary antibody (1:1000) overnight at 4°C. The washed membranes were incubated with an anti-rabbit horseradish peroxidase antibody (1:2000) for 1 h. The immunoreactive results were detected with ECL reagents and captured by a UVP BioSpectrum 500 Imaging System (Upland, CA, USA). UN-SCAN-IT gel software (Orem, UT, USA) was used to analyze and quantify the image densitometry.

### Total RNA Extraction, Real time-PCR, and PCR Analysis

Human cardiac fibroblasts were seeded on 10-cm culture dishes and treated with TNF-α. Total RNA were extracted with TRIzol reagent (Thermo Fisher, Waltham, MA, USA) according to the protocol of the manufacturer. The cDNA obtained from 0.5 μg total RNA was used to be a template for PCR amplification (TorreAmione et al., 1996). Real-time PCR was performed with KAPA PROBE FAST ABI Prism^®^ qPCR kit (KK4705, Kapa Biosystems, Wilmington, MA, USA) and 7500 Real-Time PCR System (Applied Biosystems, Foster City, CA, USA) using VCAM-1 primers and probe mixture (Forward: WSO_645213_001; Reverse: WSO_645213_002; Probe: WSO_645213_003; Invitrogen, Carlsbad, CA, USA) and endogenous GAPDH (Forward: WSO_724786_001; Reverse: WSO_724786_002; Probe: WSO_724786_003; Invitrogen) served as an internal control gene. Relative gene expression was determined by the ΔΔCt method, where Ct meant threshold cycle. All experiments were performed in triplicate. PCR were performed to analysis the expressions of NOX1 (Forward: 5′-CTTCCTCACCGGATGGGACA-3′; Reverse: 5′-TGACAGCATTTGCGCAGGCT-3′; replicon: 218 bp), NOX2 (Forward: 5′-TGTCCAAGCTGGAGTGGCAC-3′; Reverse: 5′-GCACAGCCAGTAGAAGTAGAT-3′; replicon: 321 bp), NOX3 (Forward: 5′-GAGTGGCACCCCTTCACCCT-3′; Reverse: 5′-CTAGAAGCTCTCCTTGTTGT-3′; replicon: 708 bp), Nox4 (Forward: 5′-AGTCAAACAGATGGGATA-3′; Reverse: 5′-TGTCCCATATGAGTTGTT-3′; replicon: 240 bp), Nox5 (Forward: 5′-ATGAGTGGCACCCCTTCACCATCAG-3′; Reverse: 5′-GTCAGCAGGCTCACAAACCACTCGAA-3′; replicon: 501 bp) and β-actin (Forward:5′-TGACGGGGTCACCCACACTGTGCCCATCTA-3′; Reverse: 5′-CATGAAGCATTTGCGGTGGACGATG-3′; replicon: 636 bp) was used to be the internal control.

### Plasmid Construction, Transfection, and Luciferase Reporter Gene Assays

To construct VCAM-1-Luc plasmids, human VCAM-1 promoter region from -1716 to -119 bp (kindly provided by Dr. W.C. Aird, Department of Molecular Medicine, Beth Israel Deaconess Medical Center, Boston, MA, USA), was inserted into pGL3-basic vector (Promega, Madison, WI). The AP-1 and VCAM-1-Luc transcription activities were determined as previously described (TorreAmione et al., 1996) using a luciferase assay system (Promega, Madison, WI) to analyze the firefly luciferase activities and standardize with β-galactosidase activity.

### Transient Transfection with siRNAs

HCFs (80% confluence) were washed once with PBS and 0.4 ml of serum-free DMEM/F-12 medium was added to each well. Human siRNAs of scrambled, PKCα (L-003523-00-0020) was from Dharmacon (Lafayette, CO) and Nox2 (SASI_Hs01_00086110), Nox4 (SASI_Hs02_00349918), p42 (SASI_Hs01_00058601), p38α (SASI_Hs01_00176564), JNK1 (SASI_Hs02_00319556), and c-Jun (SASI_Hs02_00333461) were from Sigma (St. Louis, MO). Transient transfection of siRNAs (100 nM) was performed using a Lipofectamine^TM^ RNAiMAX reagent according to the manufacturer’s instructions (Invitrogen, Carsbad, CA, USA).

### Measurement of Intracellular ROS Generation

The peroxide-sensitive fluorescent probe 2′,7′-dichlorofluorescein diacetate (DCF-DA) was used to assess the generation of intracellular ROS (Vesely et al., 2009) with minor modifications. HCFs were incubated with 5 μM DCF-DA in serum-free DMEM/F-12 at 37°C for 45 min. The supernatant was removed and replaced with fresh DMEM/F-12 medium before exposure to TNF-α (15 ng/ml). Relative fluorescence intensity was recorded at the indicated time by using a fluorescent plate reader (Thermo, Appliskan) at an excitation wavelength of 485 nm and emission was measured at a wavelength of 530 nm.

### Determination of NADPH Oxidase Activity by Chemiluminescence Assay

The Nox activity was examined by lucigenin chemiluminescence assay according to the previous report (Hsieh et al., 2012) with minor modification. The treated cells were gently scraped and centrifuged at 400×*g* for 10 min at 4°C. The cell pellet was resuspended in 35 μl of ice-cold PBS and kept on ice. To a final 200 μl of PBS (pre-warmed at 37°C) containing either NADPH (1 μM) or NADH (1 μM) and lucigenin (20 μM), 5 μl of cell suspension (2 × 10^4^ cells) was added to initiate the reaction followed by measurement of chemiluminescence immediately using an out-of-coincidence mode of Appliskan luminometer (Thermo^®^). Appropriate blanks and controls were established, and recorded chemiluminescence. Neither NADPH nor NADH enhanced the background chemiluminescence of lucigenin alone (30–40 counts/min). Chemiluminescence was continuously detected for 12 min, and the activity of Nox was expressed as counts per million cells.

### Cell Adhesion Assay

The human monocytic cell line THP-1 cells were obtained from ATCC (Manassas, VA, USA) and maintained in DMEM/F-12 medium containing 10% FBS at 37^o^C. Cell adhesion assay was modified from the previous report (Liu et al., 2013). In Brief, HCFs were placed on 6-well culture plates with cover slips. The cells were pretreated with the pharmacological inhibitors for 1 h and then treated with TNF-α for another 16 h at 37^o^C. THP-1 cells were maintained and suspended in DMEM/F-12 containing 10% FBS. THP-1 cells were washed, resuspended in serum-free DMEM/F-12, and incubated with BCECF/AM (10 μM, Invitrogen) for 1 h at 37°C to label the THP-1 cells. The BCECF-labeled cells were washed thrice with serum-free DMEM/F-12, resuspended in serum-free DMEM/F-12, kept in the dark at room temperature and then the labeled THP-1 cells were added to HCFs for 1 h. Gently washing the plate with serum-free DMEM/F-12 to remove the non-adherent cells, the number of adherent cells was observed by using a fluorescence microscope (ZEISS, Axiovert 200M).

### Statistical Analysis of Data

All the data were expressed as the mean or mean ± SEM of five individual experiments performed in duplicate or triplicate. All these quantified numbers (mean ± SEM) related to the group data were presented and described in Supplementary Tables S1 and S2. The significance of difference between two groups was determined by paired two-tailed Student’s *t*-test for western blot data. All others statistical analyses are comparison of multiple groups, a GraphPad Prism Program (GraphPad, San Diego, CA, USA) by one-way analysis of variance (ANOVA) followed with Tukey’s *post hoc* test has been used. A *P* < 0.05 value was considered significant.

## Results

### TNF-α Induces VCAM-1 Expression via PKCα

**FIGURE 1:**
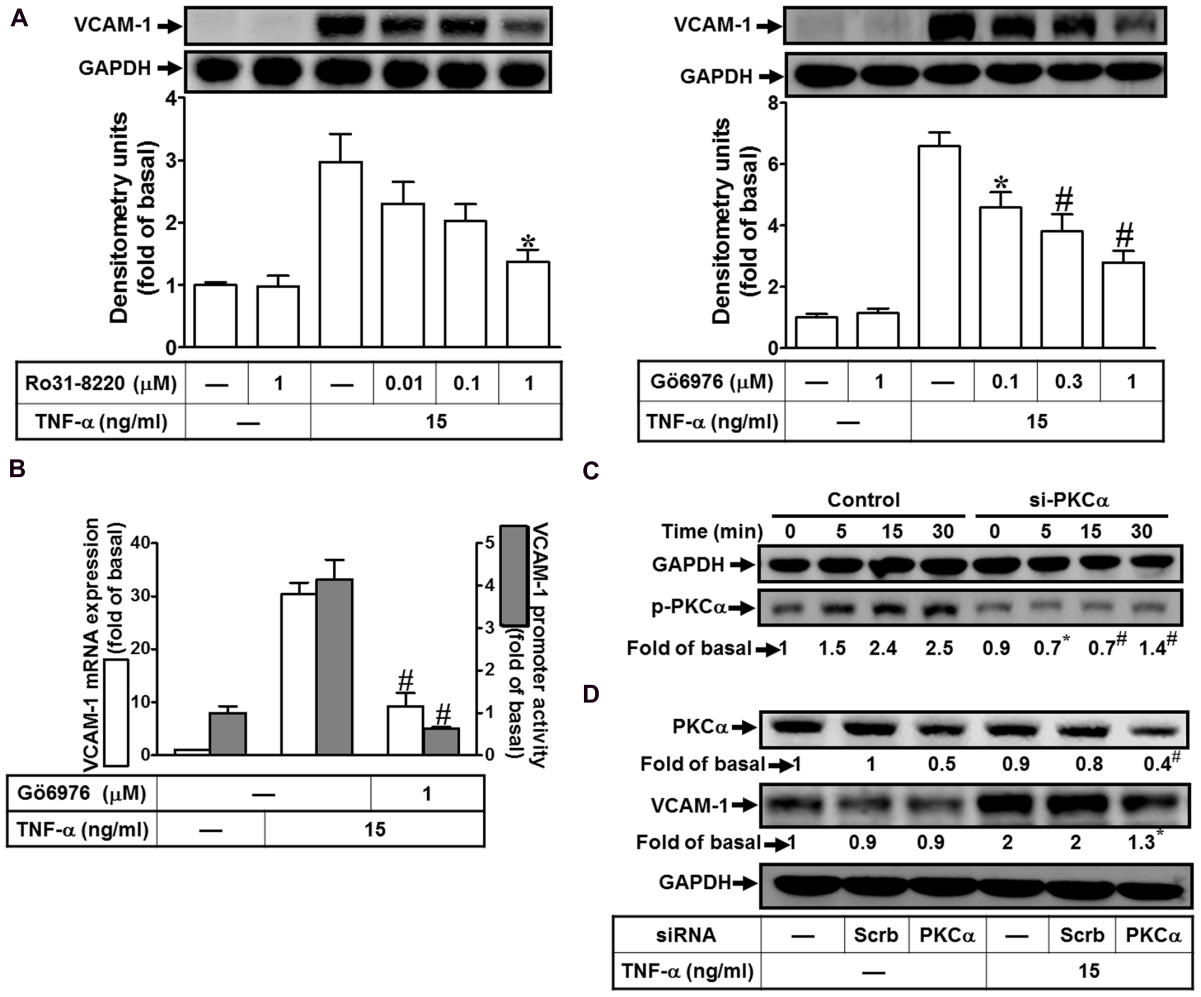
**TNF-α induces VCAM-1 expression via PKC(α)-dependent pathway in HCFs. (A)** HCFs were pretreated with Ro31-8220 (0.01, 0.1, or 1 μM) or Gö6976 (0.1, 0.3, or 1 μM) for 1 h, and then incubated with TNF-α (15 ng/ml) for 16 h. The protein levels of VCAM-1 were analyzed by Western blot. **(B)** HCFs were pretreated with Gö6976 (1 μM) for 1 h, and then incubated with TNF-α for 4 h. The mRNA expression of VCAM-1 was determined by real-time PCR (white bar). The VCAM-1 promoter activity was detected by promoter-luciferase report assay (gray bar). **(C,D)** Cells were transfected with either scrambled or PKCα siRNA, and then incubated with TNF-α for the interval time as indicated **(C)** or for 16 h **(D)**. Western blots were performed to analyze the levels of phospho-PKCα **(C)**, and total PKCα or VCAM-1 protein **(D)**. Data are expressed as mean ± SEM of three independent experiments (*n* = 3, Quantitative data of **Figures 1C,D** were presented in Supplementary Table S1). *^∗^P* < 0.05; ^#^*P* < 0.01, as compared with the cells exposed to TNF-α alone.

PKCα has been shown to regulate the expression of several proteins in various cell types (Yang et al., 2005; Lee et al., 2006; Whaley-Connell and Sowers, 2012). To determine the effects of PKCs on VCAM-1 expression, pretreatment of HCFs with either pan-PKC inhibitor Ro31-8220 or a selective PKCα inhibitor Gö6976 concentration-dependently attenuated TNF-α-induced VCAM-1 protein expression (**Figure 1A**). TNF-α-induced VCAM-1 mRNA expression and promoter activity was also inhibited by Gö6976 (1 μM) (**Figure 1B**). Next, the involvement of PKCα in TNF-α-induced responses was confirmed by determining its phosphorylation. TNF-α time-dependently stimulated PKCα phosphorylation with a maximal response within 15–30 min, which was attenuated by transfection with PKCα siRNA (**Figure 1C**, from 2.5-fold to 1.4-fold). Moreover, to ensure PKCα activity was involved in VCAM-1 expression, VCAM-1 protein was also analyzed in the PKCα siRNA transfected HCFs. We found that PKCα knockdown attenuated TNF-α-induced VCAM-1 expression about 65% (**Figure 1D**, from 2-fold to 1.3-fold). Our data suggested that the activity of PKCα transmits the TNF-α stimulation to induce VCAM-1 expression via enhancing its gene transcriptional activity in HCFs.

### ROS Involved in TNF-α-Induced VCAM-1 Expression

**FIGURE 2:**
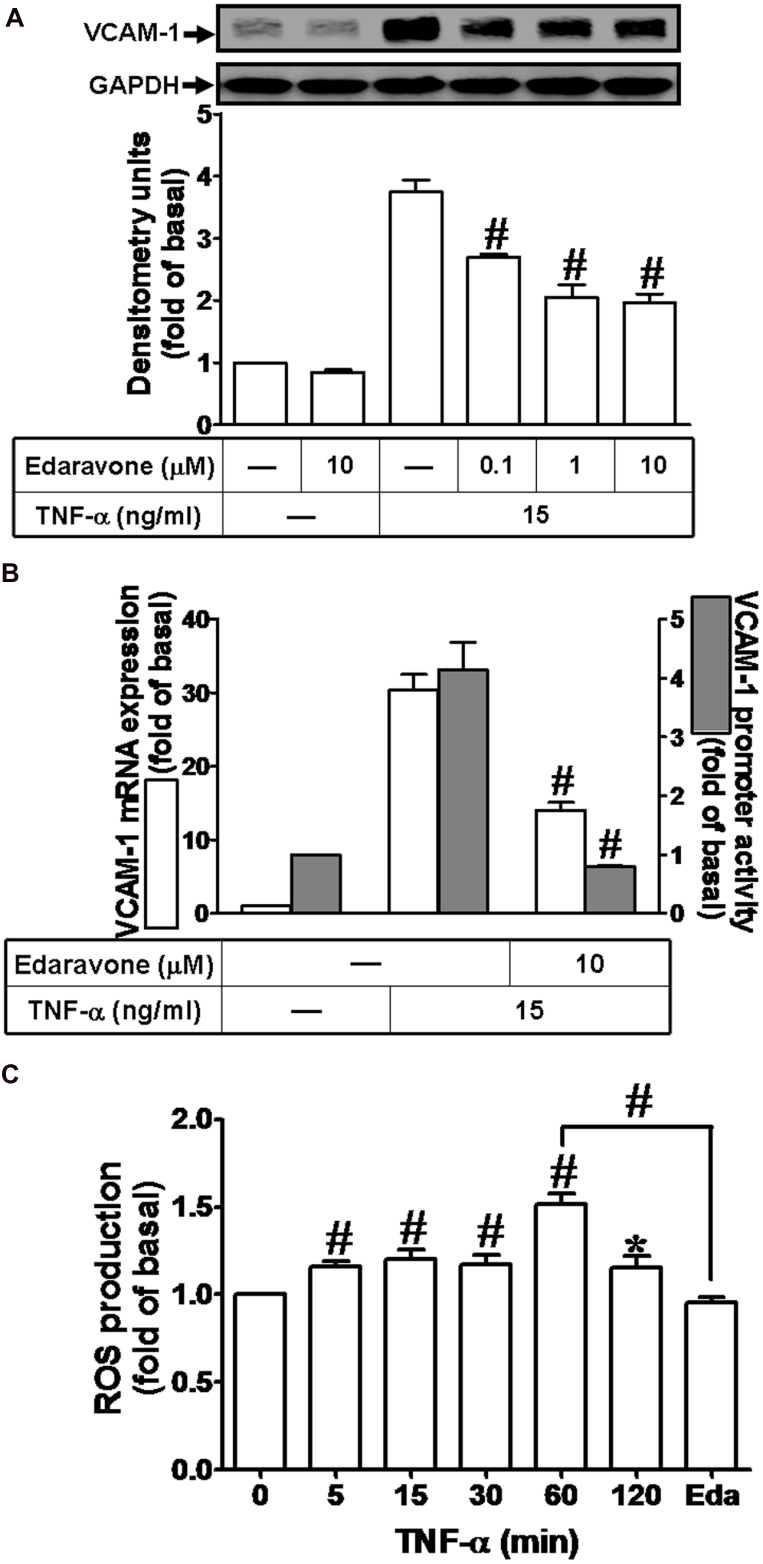
**Reactive oxygen species (ROS) signal is involved in TNF-α-induced VCAM-1 expression in HCFs. (A)** HCFs were pretreated with edaravone (0.1, 1, or 10 μM) for 1 h, and then incubated with TNF-α for 16 h. The protein levels of VCAM-1 were determined by Western blot. **(B)** HCFs were pretreated with edaravone (10 μM) for 1 h, and then incubated with TNF-α for 4 h. The mRNA expression (white bar) and promoter activity (gray bar) of VCAM-1 were determined by RT/real-time PCR or promoter report assay, respectively. **(C)** HCFs were incubated with the DCF-DA (5 μM) for 45 min, followed by pretreatment with (or without) edaravone (10 μM) and stimulation with TNF-α for the indicated time intervals or 60 min in the presence of edaravone. The fluorescence intensity of cells was determined. Data are expressed as mean ± SEM of three independent experiments (*n* = 3). *^∗^P* < 0.05; ^#^*P* < 0.01, as compared with the cells exposed to TNF-α alone for 60 min.

Previous report indicates that ROS can induce expression of adhesion molecules in various cell types (Savic-Radojevic et al., 2013). To determine whether ROS contribute to VCAM-1 induction by TNF-α in HCFs, pretreatment of Edaravone (Eda, ROS scavenger, 10 μM) was followed with TNF-α treatment. We found that Edaravone also attenuated the TNF-α-induced VCAM-1 protein expression in a concentration-dependent manner (**Figure 2A**). Besides, both of VCAM-1 mRNA expression and promoter activity induced by TNF-α were attenuated by Edaravone (10 μM) (**Figure 2B**). To evaluate the effect of TNF-α-stimulated ROS generation, as shown in **Figure 2C**, we found that TNF-α treatment time-dependently stimulated ROS generation and the maximal ROS generation was obtained within 60 min. This TNF-α-stimulated ROS generation was attenuated by pretreatment with Edaravone. These results demonstrated that Edaravone can efficiently scavenge ROS to block the VCAM-1 expression, suggesting that ROS generation contributes to TNF-α-induced VCAM-1 expression in HCFs.

### NADPH Oxidase-Derived ROS Generation Participates in TNF-α-Induced VCAM-1 Expression

**FIGURE 3:**
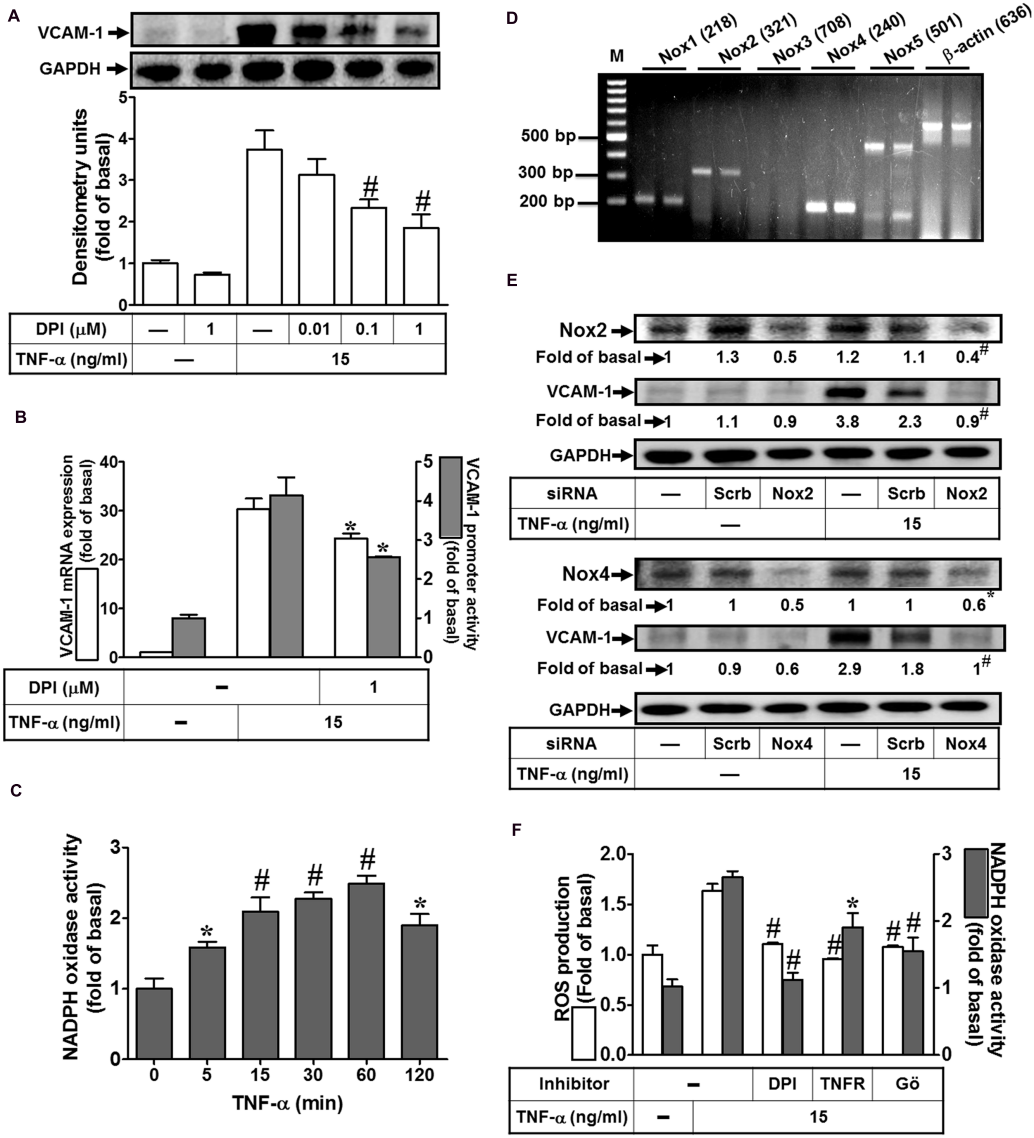
**NADPH oxidase-dependent ROS generation contributes to TNF-α-induced VCAM-1 expression in HCFs. (A)** HCFs were pretreated without or with DPI (0.01, 0.1, or 1 μM) for 1 h before exposure to TNF-α for 16 h. The protein levels of VCAM-1 were determined by Western blot. **(B)** HCFs were pretreated with DPI (1 μM) for 1 h, and then incubated with TNF-α for 4 h. The mRNA expression (white bar) and promoter activity (gray bar) of VCAM-1 were determined by RT/real-time PCR or promoter report assay, respectively. **(C)** HCFs were incubated with TNF-α for the indicated time intervals. The Nox activity was analyzed. **(D)** The mRNA expression of Nox isoforms were determined by RT-PCR analysis. **(E)** Cells were transfected with either scrambled or siRNA for Nox2 and Nox4, and then incubated with TNF-α for 16 h. The levels of Nox2, Nox4, and VCAM-1 protein were determined by Western blot. **(F)** HCFs were pretreated without or with DPI (1 μM), TNFR nAb (10 μg/ml), or Gö6976 (1 μM) for 1 h and then incubated with TNF-α for 60 min. The Nox activity (gray bar) and ROS generation (white bar) were analyzed as described in Section “Materials and Methods”. Data are expressed as mean ± SEM of three independent experiments (*n* = 3, Quantitative data of **Figures 3E,F** were presented in Supplementary Table S1). *^∗^P* < 0.05; ^#^*P* < 0.01, as compared with the cells exposed to vehicle **(C)** or TNF-α **(A,B,F)** alone.

Nox complex is considered to be a major source of cytoplasmic ROS generation implicated in physiological and pathological processes (Yokoyama et al., 1997; Yang et al., 2013b). To investigate whether TNF-α activated Nox to generate ROS leading to VCAM-1 expression, Nox inhibitor DPI was used to block the TNF-α-activated Nox activity. As shown in **Figure 3A**, we pointed out that pretreatment with DPI attenuated TNF-α-induced VCAM-1 protein expression. Moreover, both VCAM-1 mRNA expression and promoter activity induced by TNF-α were also inhibited by 1 μM DPI (**Figure 3B**). To further investigate whether TNF-α stimulates Nox activity, we found that TNF-α time-dependently stimulated Nox activity with a maximal response within 60 min in HCFs (**Figure 3C**). We also determined which Nox isoforms are expressed and involved in the TNF-α-mediated responses. RT-PCR was performed to analyze the expression of Nox isoforms in HCFs. We found that Nox1, Nox2, Nox4, and Nox5 mRNA, in particular Nox4, can be detected in HCFs (**Figure 3D**). Next, transfection with siRNAs of Nox isoforms was performed to confirm the roles of Nox(s) involved in the TNF-α-induced responses. As shown in **Figure 3E**, transfection with either Nox2 or Nox4 siRNA exhibited the consistent results that knockdown of the respective Nox protein expression can attenuate TNF-α-induced VCAM-1 expression in HCFs. Moreover, pretreatment with DPI (an inhibitor of Nox) abolished TNF-α-stimulated Nox activity and ROS generation (**Figure 3F**). To further investigate the role of TNFR-mediated PKC(α) pathway in TNF-α-stimulated Nox activity and ROS generation, pretreatment with TNFR neutralizing antibody (TNFR nAb) or Gö6976 blocked TNF-α-stimulated Nox activity and ROS generation (**Figure 3F**). These results suggested that TNF-α stimulates Nox(2/4)-derived ROS generation via TNFR-dependent PKCα cascade leading to VCAM-1 expression in HCFs.

### Involvement of p42/p44 MAPK in TNF-α-Induced VCAM-1 Expression

**FIGURE 4:**
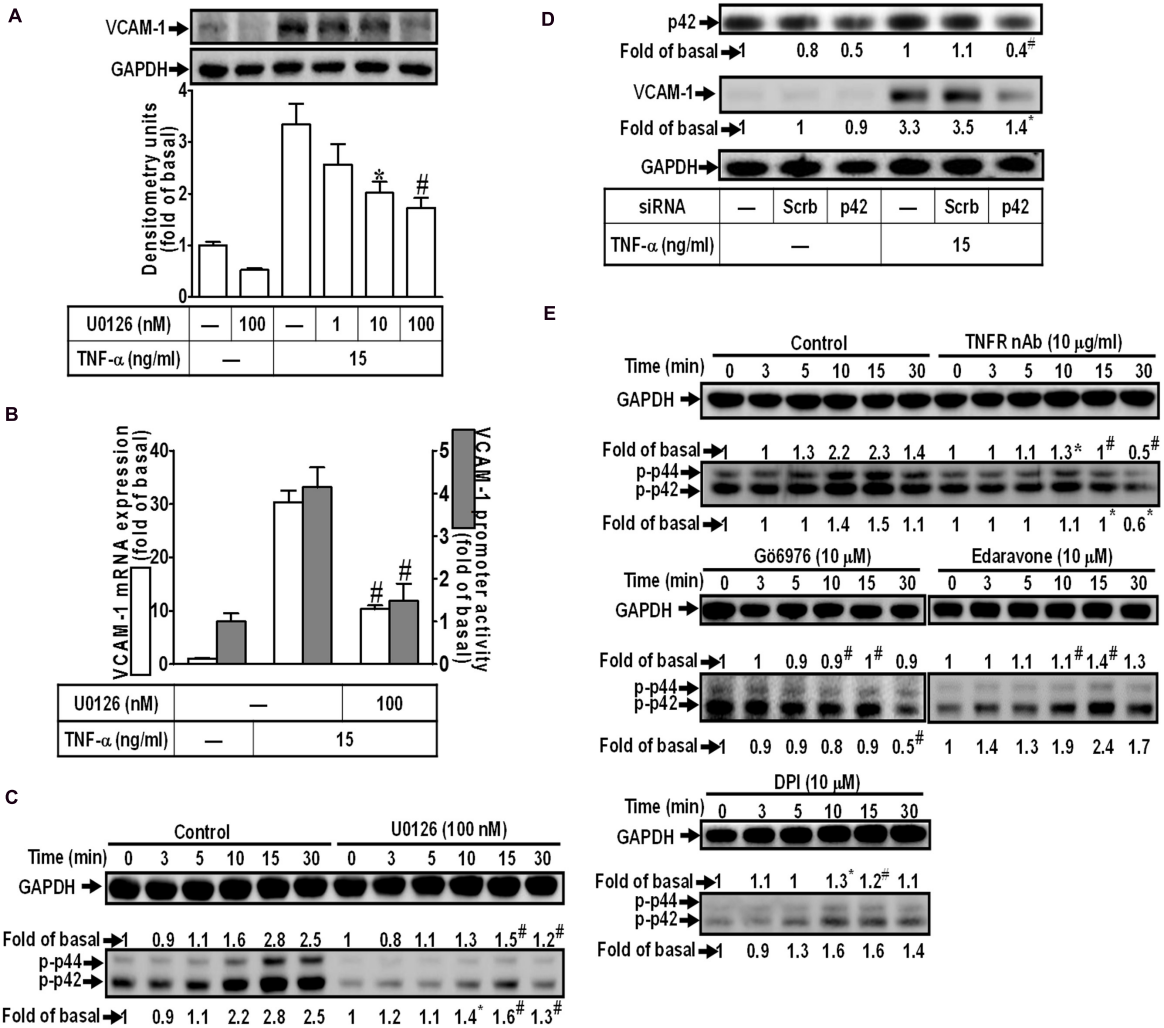
**TNF-α-induced VCAM-1 expression is mediated through p44/p42 MAPK in HCFs. (A)** HCFs were pretreated with U0126 (1, 10, or 100 nM) for 1 h, and then incubated with TNF-α for 16 h. The protein levels of VCAM-1 were determined by Western blot. **(B)** HCFs were pretreated with U0126 (100 nM) for 1 h, and then incubated with TNF-α for 4 h. The mRNA expression (white bar) and promoter activity (gray bar) of VCAM-1 were determined by real-time PCR or promoter report assay, respectively. **(C)** HCFs were pretreated with U0126 (100 nM) for 1 h, and then incubated with TNF-α for the indicated time intervals. The levels of phospho-p44/p42 MAPK were determined by Western blot. **(D)** HCFs were transfected with either scrambled or p42 siRNA, and then incubated with TNF-α for 16 h. The levels of p42 and VCAM-1 protein were determined by Western blot. **(E)** HCFs were pretreated without or with TNFR nAb (10 μg/ml), Gö6976 (10 μM), edaravone (10 μM), or DPI (10 μM) for 1 h, and then incubated with TNF-α for the indicated time intervals. The levels of phospho-p44/p42 MAPK were determined by Western blot. Data are expressed as mean ± SEM of three independent experiments (*n* = 3, Quantitative data of **Figures 4C–E** were presented in Supplementary Table S1). *^∗^P* < 0.05; ^#^*P* < 0.01, as compared with the cells exposed to TNF-α alone.

Activation of MAPKs plays a key role in the pathogenesis of heart diseases such as myocardial hypertrophy (Rajesh et al., 2003; Palomer et al., 2013). To determine the role of p42/p44 MAPK in VCAM-1 expression, pretreatment with a MEK1/2 inhibitor U0126 concentration-dependently attenuated TNF-α-induced VCAM-1 expression (**Figure 4A**). U0126 (100 nM) also repressed TNF-α-induced VCAM-1 mRNA expression and transcription activity (**Figure 4B**). To investigate whether TNF-α-induced responses are mediated through activation of p42/p44 MAPK. The results of **Figure 4C** indicated that TNF-α treatment time-dependently stimulated p42/p44 MAPK phosphorylation with a maximal response within 10–15 min, which was attenuated by U0126 (100 nM). To further ensure the role of p42/p44 MAPK in regulation of VCAM-1-expression, p42 MAPK siRNA transfection was used to knock down p42 protein. We found that knockdown of p42 protein by p42 siRNA transfection also attenuated TNF-α-induced VCAM-1 expression (**Figure 4D**). In addition, pretreatment with not only U0126, but also TNFR nAb (10 μg/ml), Gö6976 (10 μM), Edaravone (10 μM), and DPI (10 μM), could attenuate TNF-α-stimulated p42/p44 MAPK phosphorylation (**Figure 4E**). These results suggested that TNF-α triggers the p42/p44 MAPK phosphorylation leading to VCAM-1 induction via a TNFR-mediated PKCα/Nox/ROS pathway in HCFs.

### Involvement of p38 MAPK in TNF-α-Induced VCAM-1 Expression

**FIGURE 5:**
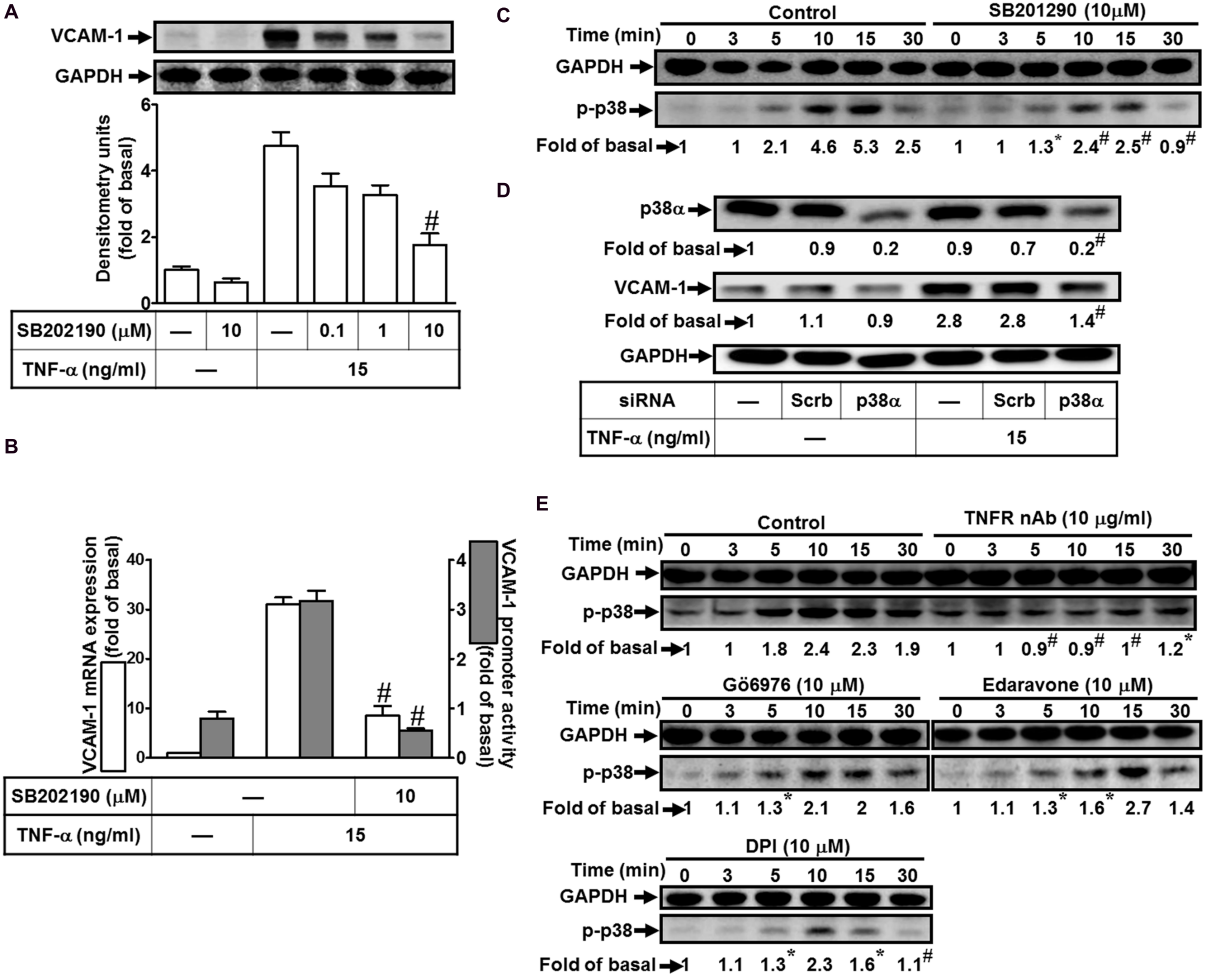
**Involvement of p38 MAPK in TNF-α-induced VCAM-1 expression in HCFs. (A)** HCFs were pretreated with SB202190 (0.1, 1, or 10 μM) for 1 h, and then incubated with TNF-α for 16 h. The protein levels of VCAM-1 were determined by Western blot. **(B)** Cells were pretreated with SB202190 (10 μM) for 1 h, and then incubated with TNF-α for 4 h. The mRNA expression (white bar) and promoter activity (gray bar) of VCAM-1 were determined by real-time PCR or promoter report assay, respectively. **(C)** Cells were pretreated with SB202190 (10 μM) for 1 h, and then incubated with TNF-α for the indicated time intervals. The levels of phospho-p38 MAPK were determined by Western blot. **(D)** Cells were transfected with either scrambled or p38 MAPK siRNA, and then incubated with TNF-α for 16 h. The levels of p38 MAPK and VCAM-1 protein were determined by Western blot. **(E)** Cells were pretreated without or with TNFR nAb (10 μg/ml), Gö6976 (10 μM), edaravone (10 μM), or DPI (10 μM) for 1 h, and then incubated with TNF-α for the indicated time intervals. The levels of phospho-p38 MAPK were determined by Western blot. Data are expressed as mean ± SEM of three independent experiments (*n* = 3, Quantitative data of **Figures 5C–E** were presented in Supplementary Table S1). ^#^*P* < 0.01, as compared with the cells exposed to TNF-α alone.

To investigate the effect of p38 MAPK on VCAM-1 induction, pretreatment with a p38 MAPK inhibitor SB202190 concentration-dependently attenuated TNF-α-induced VCAM-1 expression (**Figure 5A**). Pretreatment with SB202190 (10 μM) also attenuated TNF-α-induced VCAM-1 mRNA expression and transcription activity (**Figure 5B**). To determine whether the activation of p38 MAPK was required for TNF-α-mediated responses, as shown in **Figure 5C**, TNF-α time-dependently stimulated phosphorylation of p38 MAPK with a maximal response within 10 min, which was prevented by SB202190 (10 μM). To further confirm the role of p38 MAPK in TNF-α-induced VCAM-1 expression, transfection with p38 MAPK siRNA to knock down p38 protein. We observed that knockdown of p38 protein also attenuated TNF-α-induced VCAM-1 expression (**Figure 5D**). Moreover, phosphorylation of p38 MAPK stimulated by TNF-α was also attenuated by pretreatment with TNFR nAb (10 μg/ml), Gö6976 (10 μM), Edaravone (10 μM), or DPI (10 μM) (**Figure 5E**). These results suggested that TNF-α activates TNFR/PKCα/Nox/ROS linking to p38 MAPK phosphorylation and leading to VCAM-1 expression in HCFs.

### Involvement of JNK1/2 in TNF-α-Induced VCAM-1 Expression

**FIGURE 6:**
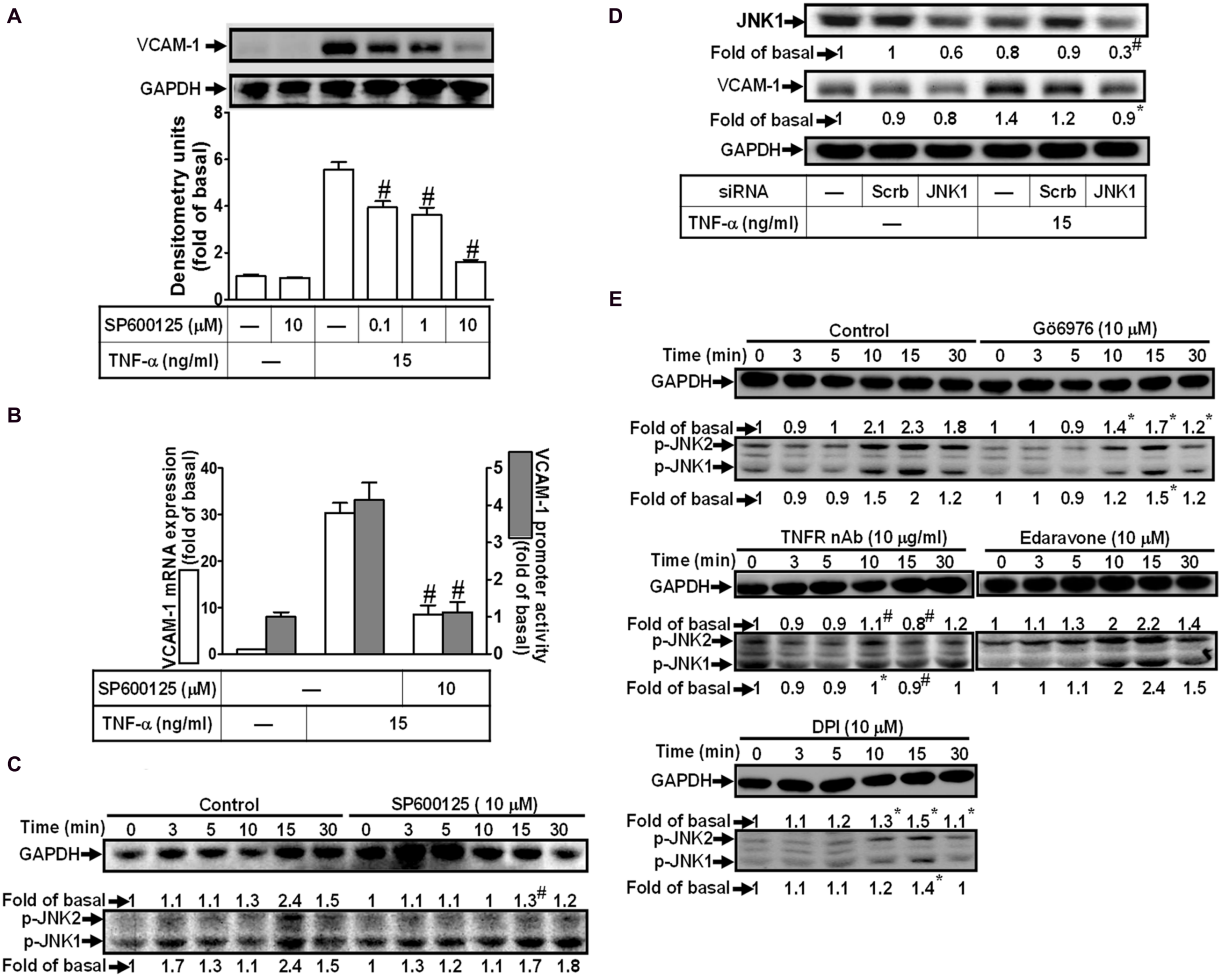
**Involvement of JNK1/2 in TNF-α-induced VCAM-1 expression in HCFs. (A)** HCFs were pretreated with SP600125 (0.1, 1, or 10 μM) for 1 h, and then incubated with TNF-α for 16 h. The protein levels of VCAM-1 were determined by Western blot. **(B)** HCFs were pretreated with SP600125 (10 μM) for 1 h, and then incubated with TNF-α for 4 h. The mRNA expression (white bar) and promoter activity (gray bar) of VCAM-1 were determined by real-time PCR or promoter report assay, respectively. **(C)** HCFs were pretreated with SP600125 (10 μM) for 1 h, and then incubated with TNF-α for the indicated time intervals. The levels of phospho-JNK1/2 were determined by Western blot. **(D)** HCFs were transfected with either scrambled or JNK1 siRNA, and then incubated with TNF-α for 16 h. The levels of JNK1 and VCAM-1 protein were determined by Western blot. **(E)** HCFs were pretreated without or with TNFR nAb (10 μg/ml), Gö6976 (10 μM), edaravone (10 μM), or DPI (10 μM) for 1 h, and then incubated with TNF-α for the indicated time intervals. The levels of phospho-JNK1/2 were determined by Western blot. Data are expressed as mean ± SEM of three independent experiments (*n* = 3, Quantitative data of **Figures 6C–E** were presented in Supplementary Table S2). ^#^*P* < 0.01, as compared with the cells exposed to TNF-α alone.

To determine the role of JNK1/2 signaling in VCAM-1 expression, pretreatment with JNK1/2 inhibitor (SP600125) concentration-dependently attenuated TNF-α-induced VCAM-1 expression (**Figure 6A**). TNF-α-induced VCAM-1 mRNA expression and transcription activity was also attenuated by pretreatment with SP600125 (10 μM) (**Figure 6B**). We further determined whether TNF-α-stimulated JNK1/2 activation is required for these responses, the activation of JNK1/2 was determined by Western blot using an anti-phospho-JNK1/2 antibody. As shown in **Figure 6C**, TNF-α time-dependently stimulated JNK1/2 phosphorylation with a maximal response within 15 min, which was attenuated by pretreatment with SP600125 (10 μM). To ensure the role of JNK1/2 in VCAM-1 expression, as shown in **Figure 6D**, transfection with JNK1 siRNA knocked down JNK1 protein and also attenuated TNF-α-induced VCAM-1 expression. Moreover, TNF-α-stimulated JNK1/2 phosphorylation was also attenuated by pretreatment with TNFR nAb (10 μg/ml), Gö6976 (10 μM), Edaravone (10 μM), or DPI (10 μM) (**Figure 6E**). These results suggested that TNF-α-induced VCAM-1 expression is mediated through TNFR-dependent PKCα/Nox/ROS pathway linking to JNK1/2 phosphorylation in HCFs.

### AP-1 Involved in TNF-α-Induced VCAM-1 Expression

**FIGURE 7:**
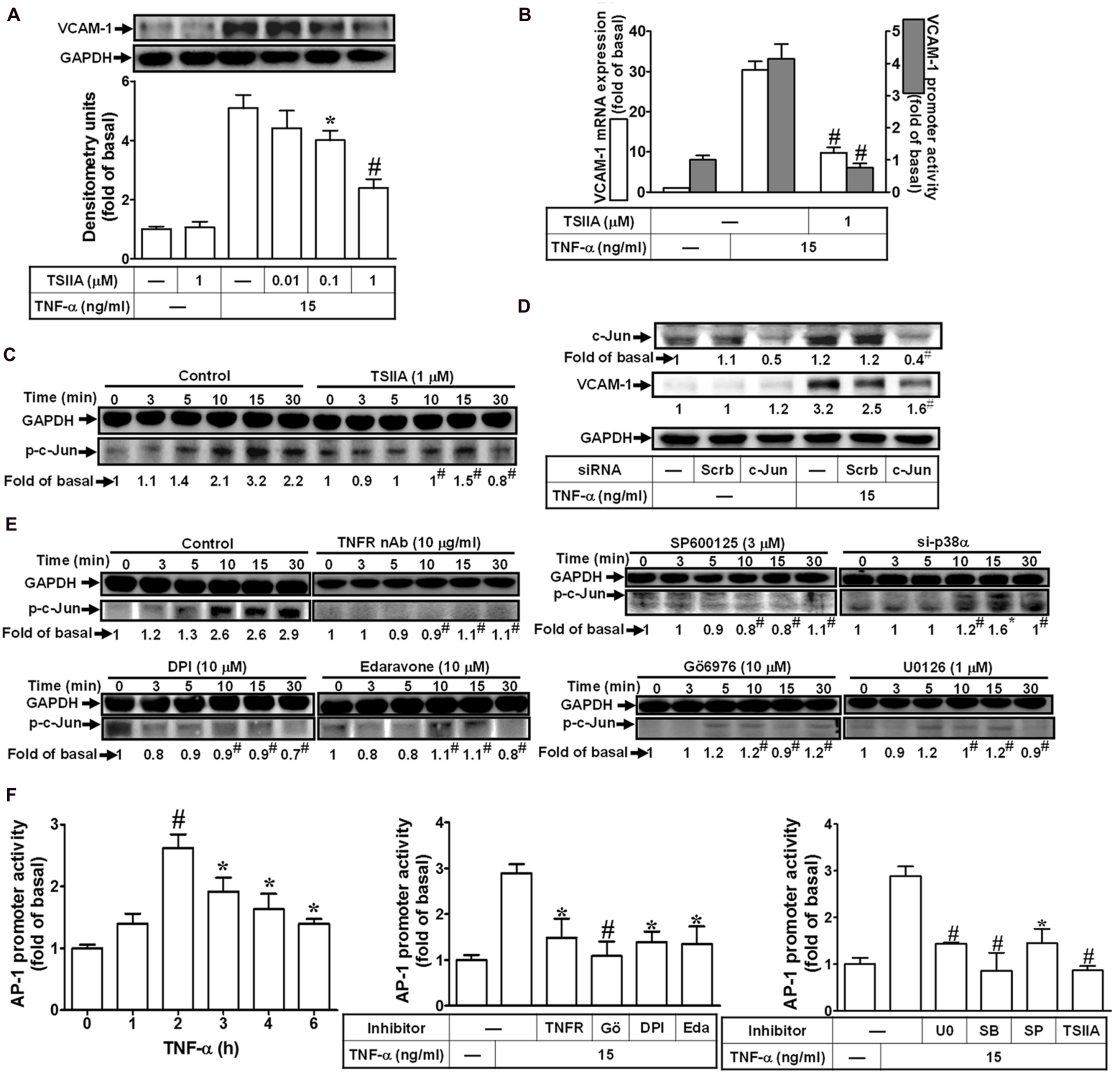
**Activation of c-Jun/AP-1 is required for TNF-α-mediated VCAM-1 expression. (A)** HCFs were pretreated with TSIIA (0.01, 0.1, or 1 μM) for 1 h, and then incubated with TNF-α for 16 h. The protein levels of VCAM-1 were determined by Western blot. **(B)** HCFs were pretreated with TSIIA (1 μM) for 1 h, and then incubated with TNF-α for 4 h. The mRNA expression (white bar) and promoter activity (gray bar) of VCAM-1 were determined by real-time PCR or promoter report assay, respectively. **(C)** HCFs were pretreated with TSIIA (1 μM) for 1 h, and then incubated with TNF-α for the indicated time intervals. The levels of phospho-c-Jun were determined by Western blot. **(D)** HCFs were transfected with either scrambled or c-Jun siRNA, and then incubated with TNF-α for 16 h. The levels of c-Jun and VCAM-1 protein were determined by Western blot. **(E)** HCFs were pretreated without or with TNFR nAb (10 μg/ml), Gö6976 (10 μM), edaravone (10 μM), DPI (10 μM), U0126 (1 μM), SP600125 (3 μM) for 1 h, or p38 siRNA transfection, and then incubated with TNF-α for the indicated time intervals. The levels of phospho-c-Jun were determined by Western blot. **(F)** HCFs were transiently cotransfected with pAP1-Luc and pCMV-Gal for 24 h, and then incubated with TNF-α for the indicated time intervals (**F**, left). The transfected cells were pretreated with TNFR nAb, Gö6976, DPI, edaravone (Eda) (**F**, middle), apocynin (APO), SB202190 (SB), SP600125 (SP), or TSIIA (**F**, right) for 1 h and then incubated with TNF-α for 2 h. The AP-1 transcription activity in the cell was determined as described in Section “Materials and Methods”. Data are expressed as mean ± SEM of three independent experiments (*n* = 3, Quantitative data of **Figures 7C–E** were presented in Supplementary Table S2). *^∗^P* < 0.05; ^#^*P* < 0.01, as compared with the cells exposed to TNF-α alone.

AP-1, heterodimer of c-Fos and c-Jun, is a well-known transcription factor to participate in the expression of inflammatory genes in various cell types (Hsieh and Yang, 2013). To determine whether AP-1 is involved in VCAM-1 expression, the AP-1 inhibitor, TSIIA, was used to block the TNF-α-induced VCAM-1 expression. We found that pretreatment with TSIIA obviously inhibited the TNF-α-induced VCAM-1 expression in a concentration-dependent manner (**Figure 7A**). Consistently, both of the TNF-α-induced VCAM-1 mRNA expression and promoter activity were also attenuated with pretreatment of TSIIA (1 μM) (**Figure 7B**). We further determine whether TNF-α stimulated AP-1 activation required for VCAM-1 expression, the activated AP-1 was detected by Western blot using an anti-phospho-c-Jun antibody. As shown in **Figure 7C**, we found that TNF-α obviously stimulated c-Jun phosphorylation with a maximal response within 15 min, which was also inhibited by pretreatment with TSIIA (1 μM). To further ensure c-Jun/AP-1 involved in VCAM-1 expression, c-Jun siRNA transfection was used to knock down c-Jun protein expression. We found that knockdown of c-Jun protein also attenuated TNF-α-induced VCAM-1 expression (**Figure 7D**). Moreover, we also differentiated the signaling components involved in TNF-α-stimulated the phosphorylation of c-Jun/AP-1. The results of Western blot indicated that pretreatment with TNFR nAb, Edaravone or DPI attenuated TNF-α-stimulated c-Jun phosphorylation. Next, to investigate whether activation of MAPKs led to c-Jun phosphorylation, we found that pretreatment with Gö6976 (10 μM), U0126 (1 μM), or SP600125 (3 μM) and p38 siRNA transfection also inhibited TNF-α-stimulated c-Jun phosphorylation (**Figure 7E**). Furthermore, TNF-α time-dependently enhanced AP-1 transcription activity with a maximal response within 2 h (**Figure 7F**, left), which was also attenuated by pretreatment with TNFR nAb or pharmacological inhibitors including Gö6976, DPI, Edaravone, U0126, SB202190, SP600125, or TSIIA (**Figure 7F**, middle and right). These results suggested that TNF-α-stimulated c-Jun/AP-1 activation via TNFR-mediated PKCα/Nox/ROS/MAPKs is required for up-regulation of VCAM-1 in HCFs.

### TNF-α Promotes Monocytes Adhesion Through an AP-1-Dependent Up-Regulation of VCAM-1

**FIGURE 8:**
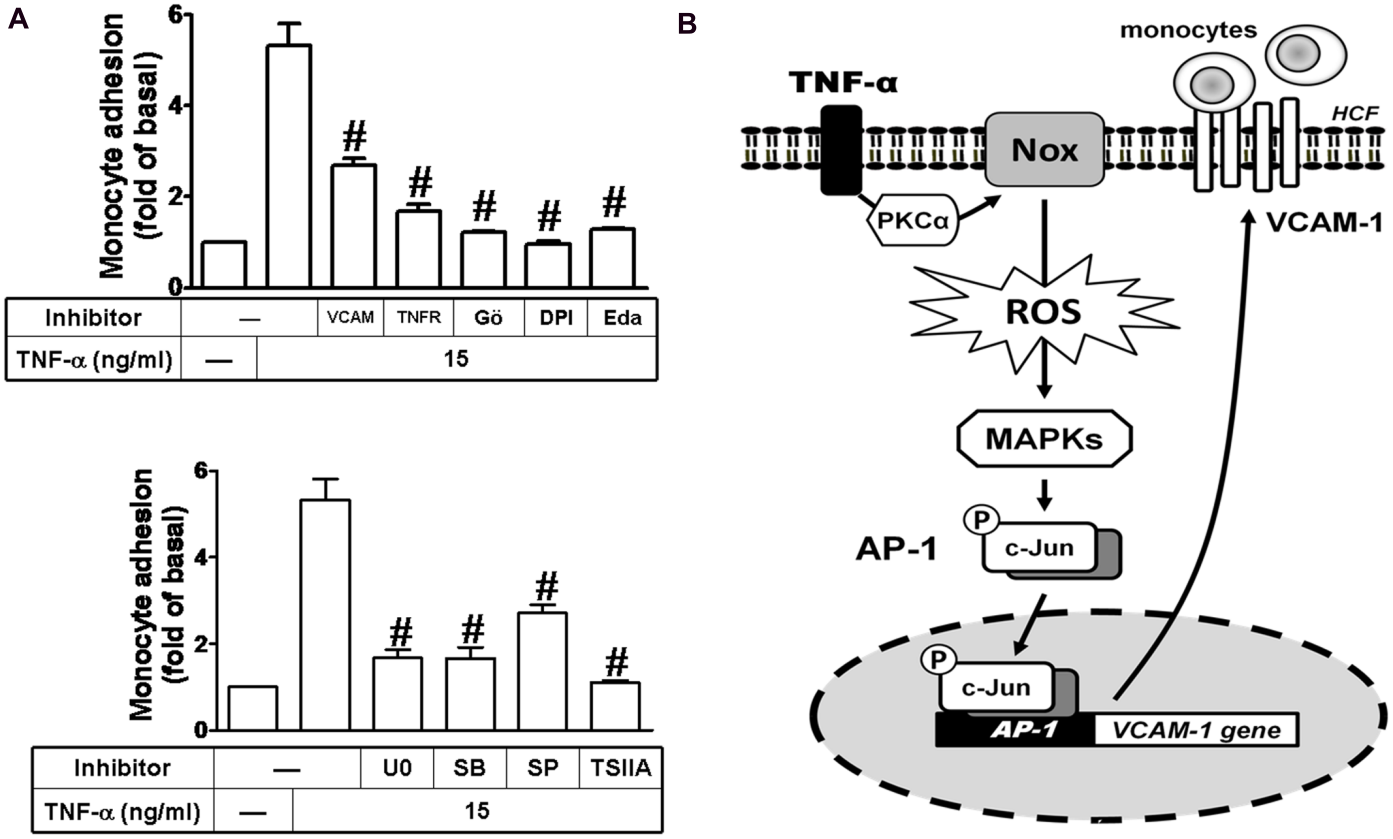
**Up-regulated VCAM-1 by TNF-α contributes to promotion of monocyte adhering to these HCFs. (A)** For cell adhesion activity, cells were pretreated with VCAM-1 nAb, TNFR nAb, Gö6976 (Gö), DPI, edaravone (Eda) (**Figure 7A**, upper), U0126 (U0), SB202190 (SB), SP600125 (SP), or TSIIA (**Figure 7A**, lower) for 1 h and following by TNF-α (15 μg/ml) for 16 h prior to addition of THP-1 monocytes. The adhesion activity was determined by cell adhesion assay as described in Section “Materials and Methods”. Data are expressed as mean ± SEM of at least five individual experiments (*n* = 5). ^#^*P* < 0.01 as compared with TNF-α alone. **(B)** Schematic signaling pathways are involved in TNF-α-induced VCAM-1 expression in HCFs. TNF-α-induced VCAM-1 expression is triggered by TNF-α/TNFR-mediated signaling pathways. TNF-α-mediated responses are dependent on PKC-α-activated Nox/ROS/MAPKs cascades linking to c-Jun/AP-1 transcription activity. Up-regulation of VCAM-1 contributes to enhancement of monocyte adhering to these HCFs challenged with TNF-α. Understanding the mechanisms of VCAM-1 up-regulated by TNF-α on HCFs may provide rationally therapeutic interventions for heart injury or inflammatory diseases.

Ultimately, we investigated the functional activity of up-regulated VCAM-1 by TNF-α in HCFs. Previous reports indicate that THP-1 monocytes can be used as a model system to mimetic the adhesion of monocytes to upregulated adhesion molecules on cell surface of resident cells induced by various stimuli (Goda et al., 2000; Wu et al., 2010; Liu et al., 2013). In this report, THP-1 adhesion assay was performed to evaluate the functional activity of VCAM-1 expression in TNF-α-challenged HCFs. Our results indicated that treatment of HCFs with TNF-α significantly increased the adhesion of THP-1 monocyte to HCFs (∼5-folds) which was attenuated by pretreatment with VCAM-1 nAb or TNFR nAb (**Figure 8A**, upper). As mentioned above, pretreatment with DPI, Gö6976, Edaravone, U0126, SB202190, SP600125, and TSIIA attenuated the TNF-α-induced VCAM-1 expression, consistently, the number of monocytes attached to TNF-α-challenged HCFs was also reduced by these pharmacological inhibitors (**Figure 8A**). These results suggested that up-regulation of VCAM-1 via TNFR-mediated PKCα/Nox/ROS/MAPKs/AP-1 pathway plays a critical role to promote monocytes adhesion to TNF-α-challenged HCFs.

## Discussion

Up-regulation of adhesion molecules on the surface of the heart cells plays a key role in the recruitment and infiltration of leukocytes at sites of inflammation in heart inflammatory disorders (Bedard and Krause, 2007; Cheng et al., 2009; Cook-Mills et al., 2011). TNF-α has been confirmed to induce VCAM-1 expression in various cell types such as synovial fibroblasts (TorreAmione et al., 1996), but the intracellular signaling mechanisms remain unclear in HCFs. Moreover, TNF-α has also been shown to activate various signaling molecules including protein kinases (e.g., PKCs), ROS, and MAPKs in several cell types (TorreAmione et al., 1996; Lin et al., 2012; Chi et al., 2014). Herein, we investigate detail mechanisms underlying TNF-α-induced VCAM-1 expression in HCFs and its effects on monocytes adhesion. We found that in HCFs, TNF-α stimulates PKCα-dependent Nox/ROS production via TNFR1, in turn initiates the activation of MAPK pathway and to accelerate AP-1 phosphorylation. The AP-1 activity leads to enhancing VCAM-1 promoter activity and then up-regulating VCAM-1 protein in these cells. In addition, the upregulation of VCAM-1 was obviously correlated with monocytes adhesion to HCFs challenged with TNF-α.

Excessive production of ROS has been shown to play a causative role in numerous pathologies of heart diseases (Yokoyama et al., 1997). ROS also exert as an internal messenger to maintain normal physiological functions or the side products of inflammatory responses in a concentration-dependent manner (Lin et al., 2009). When exposed to oxidative stress, excessive production of ROS may trigger deleterious effects within the heart. It has been shown that activated renal mesangial cells produce large amounts of ROS which can lead to impairing cellular functions and enhancing inflammatory reactions (Lee et al., 2012). These immune cells are stimulated when encountering inhaled microorganisms or other mediators leading to the activation of Nox and the production of ROS. It has been reported that Nox activation is involved in the regulation of VCAM-1 expression and intracellular ROS generation induced by various stimuli such as LPS and ammonia (Reinehr et al., 2007; Lee et al., 2012). Therefore, we explored that the roles of Nox-dependent ROS generation in HCFs is associated with VCAM-1 expression in response to TNF-α. First, a potent and novel scavenger of free radicals, Edaravone (Saito et al., 2012), was used to scavenge ROS including hydroxyl radicals. Edaravone has been shown to protect against mitochondrial injury in myocardial ischemia/reperfusion and inhibits inflammatory responses (Kono et al., 2003; Rajesh et al., 2003). In the present study, edaravone significantly inhibited the TNF-α-induced ROS generation and VCAM-1 protein, mRNA, and promoter activity, suggesting that ROS signal is critical to TNF-α-induced VCAM-1 expression. Next, DPI, a Nox inhibitor, was used to confirm whether Nox-derived ROS generation is involved in the response. Our results showed that pretreatment with DPI attenuated TNF-α-induced ROS generation and VCAM-1 expression, suggesting that Nox plays an important role in these responses. We further demonstrated that two Nox isoforms, including Nox2 and Nox4, contribute to TNF-α-induced VCAM-1 expression by transfection with siRNA of Nox2 or Nox4. These data are consistent with previous reports showing that Nox-derived ROS generation is involved in VCAM-1 induction by IL-1β in HTSMCs (Luo et al., 2009) or human renal mesangial cells (Lee et al., 2012). In addition, we also demonstrated that PKC(α) is an upstream molecule of Nox-derived ROS generation by using the inhibitor or siRNA of PKCα, consistent with the study indicating that PKCα is involved in VCAM-1 expression in HTSMCs (Lee et al., 2008) and cPLA_2_ expression in RA synovial fibroblasts (Chi et al., 2014). These results suggested that PKCα contributes to TNF-α-induced Nox/ROS-mediated VCAM-1 expression in HCFs.

Abnormal MAPK regulation might lead to various inflammation and injury in the heart diseases (Hsieh et al., 2012; Palomer et al., 2013). Among the MAPK family, ERK1/2, p38 MAPK, and JNK1/2 play critical roles in signaling pathways that regulate cell growth and inflammation. These MAPK signaling components were associated with the effects of pro-inflammatory mediators such as TNF-α (Lee et al., 2006; Lin et al., 2012). In the present study, we demonstrated that ERK1/2, p38 MAPK, and JNK1/2 are essentially required for VCAM-1 induction by TNF-α, since TNF-α-induced responses were attenuated by their respective pharmacological inhibitors or transfection with siRNA. The involvement of MAPKs in VCAM-1 induced by TNF-α was further confirmed to analyze the phosphorylation of ERK1/2, p38 MAPK, and JNK1/2. These findings were consistent with our previous reports that VCAM-1 expression was mediated through activation of ERK1/2, p38 MAPK, and JNK1/2 in TNF-α treated HTSMCs (Lee et al., 2006) and activation of p38 MAPK by LPS in human renal mesangial cells (Reinehr et al., 2007). Moreover, TNF-α-stimulated phosphorylation of ERK1/2, p38 MAPK, and JNK1/2 was significantly attenuated by pretreatments with TNFR nAb, PKCα inhibitors (Gö6976), ROS scavenger (edaravone), or Nox inhibitor (DPI and APO), suggesting that PKCα-dependent Nox/ROS signals are crucial for TNF-α-bound-TNFR-mediated MAPKs phosphorylation in HCFs. Our results were consistent with the previous reports showing that ROS-dependent MAPKs activity is involved in the regulation of cellular functions by diverse stimuli in several cell types (Reinehr et al., 2007; Cheng et al., 2009). External stimuli activate distinct groups of MAPKs by a ROS-dependent manner which may exhibit the cell type-specificity.

The progression of oxidative stress during cellular or tissue injuries not only causes damage to cellular macromolecules by oxidation, but also alters functional activity of transcription factors to modulate the pattern of gene expression. Here we focus on the roles of transcription factor AP-1, the ROS-dependent AP-1 activity which is well-known to modulate gene transcriptions during oxidative stress associated with inflammatory diseases (Sen and Packer, 1996). To investigate the role of AP-1 in these TNF-α-mediated responses, our results further indicated that TNF-α stimulates phosphorylation of c-Jun, an AP-1 subunit, via PKCα-mediated Nox/ROS linking to MAPK pathway. Moreover, c-Jun/AP-1 plays the terminal acceptor of signal transduction in TNF-α-induced VCAM-1 expression. Thus, to knockdown the AP-1 activity with c-Jun siRNA transfection also prevents the TNF-α-driven VCAM-1 expression. Previous reports also provide evidence indicating that the upstream region of VCAM-1 promoter contains a variety of putative responsible elements including AP-1 consensus sequence in several cell types (Vesely et al., 2009; Lin et al., 2012). Thus, we also confirmed that TNF-α-induced VCAM-1 promoter activity was mediated through a TNFR-dependent PKCα-dependent Nox/ROS/MAPK/AP-1 pathway. These results are consistent with the reports indicating that LPS triggers the activity of PKCs initiated the subsequent activation of the MAPK pathways and AP-1 phosphorylation to induce VCAM-1 expression in HTSMCs (Lin et al., 2009) and human renal mesangial cells (Reinehr et al., 2007).

The infiltration of immune cells is due to up-regulation of adhesion molecules such as VCAM-1 (Bedard and Krause, 2007; Cheng et al., 2009; Cook-Mills et al., 2011), which contributes to chronic inflammatory diseases including cardiovascular diseases. The level of VCAM-1 is low on unstimulated endothelial cells, which can be up-regulated after challenge with cytokines. In the present study, we demonstrated that TNF-α-enhanced monocyte (THP-1) adhesion through up-regulation of VCAM-1 on HCFs was attenuated by VCAM-1 nAb. Moreover, these signaling components implicated in VCAM-1 induction by TNF-α were also correlated with TNF-α-enhanced monocyte adhesion confirmed by using respective pharmacological inhibitors. These results indicated that TNF-α-induced VCAM-1 expression via PKCα-dependent activation of Nox/ROS/MAPKs linking to AP-1 pathway, which results in monocytes adhered to the TNF-α-challenged HCFs. These results are consistent with a report indicating that TNF-α enhanced immune cells adhesion via up-regulation of VCAM-1 in HTSMCs (Lee et al., 2006) and RASFs.

## Conclusion

Based on the observations from literatures and our findings, we proposed a VCAM-1 gene expression model as shown in **Figure 8B** to depict the implicated components of signaling mechanisms in the TNF-α-treated HCFs. These findings imply that because TNF-α triggers the signal transductions of PKCα-mediated activation of Nox/ROS, MAPKs, and AP-1 to up-regulation of VCAM-1 in HCFs, the expression of adhesion molecules could enhance the attachment and linkage of monocytes to HCFs. The results provide new insights to understand the mechanisms by which TNF-α stimulated VCAM-1 expression in HCFs associated with amplifying the inflammatory responses, supporting the hypothesis that TNF-α may play a key role in the exaggeration of cardiac diseases.

## Author Contributions

CCL, CCY, CYW, HCT, CSP, LDH, and CMY substantially contributed to the conception or design of the work, the acquisition, analysis, and interpretation of data for the work. CCL, CCY, CYW, HCT, CSP, LDH, and CMY drafted the work and revised it critically for important intellectual content. CCL, CCY, CYW, HCT, CSP, LDH, and CMY finally approved the version to be published. CCL, CCY, CYW, HCT, CSP, LDH, and CMY agreed to be accountable for all aspects of the work in ensuring that questions related to the accuracy or integrity of any part of the work are appropriately investigated and resolved.

## Conflict of Interest Statement

The authors declare that the research was conducted in the absence of any commercial or financial relationships that could be construed as a potential conflict of interest.
