# Supplementary material for: NADPH Oxidase/ROS-Dependent VCAM-1 Induction on TNF-α-Challenged Human Cardiac Fibroblasts Enhances Monocyte Adhesion
Source: Front Pharmacol. 2016 Jan 28;6:310. doi: 10.3389/fphar.2015.00310 (PMC4729888; doi:10.3389/fphar.2015.00310)
Supplement: Supplementary file 1 [file Table_1.PDF]

Supplementary Table 1

Figure 1C.

	Control				Si-PKCα				N
Time (min)	0	5	15	30	0	5	15	30	
p-PKCα (Fold of basal)	1.0	1.5 ±0.04	2.4 ±0.2	2.5 ±0. 4	0.9 ±0.03	0.7 ±0.06	0.7 ±0.02	1.4 ±0.34	3

Figure 1D.

siRNA	—	Scrb	PKCα	—	Scrb	PKCα	N
TNF-α	—			15 ng/ml			
PKCα (Fold of basal)	1.0	1.0±0.04	0.5±0.05	0.9±0.23	0.8±0.17	0.4±0.17	3
VCAM-1 (Fold of basal)	1.0	0.9±0.11	0.9±0.08	2.0±0.03	2.0 ±0.07	1.3±0.08	3

Figure 3E.

siRNA	—	Scrb	Nox2	—	Scrb	Nox2	N
TNF-α	—			15 ng/ml			
Nox2 (Fold of basal)	1.0	1.3±0.2	0.5±0.08	1.2±0.03	1.1±0.25	0.4±0.07	3
VCAM-1 (Fold of basal)	1.0	1 ±0.41	0.9 ±0.38	3.8±0.49	2.3 ±0.37	0.9 ±0.21	3

siRNA	—	Scrb	Nox4	—	Scrb	Nox4	N
TNF-α	—			15 ng/ml			
Nox4 (Fold of basal)	1.0	1.3±0.02	0.5±0.01	1.0±0.13	1.0±0.25	0.6±0.04	3
VCAM-1 (Fold of basal)	1.0	0.9 ±0.15	0.6 ±0.18	2.9±0.34	1.8 ±0.47	1.0 ±0.05	3

Figure 4C.

Treatment	Control						U0126						N
Time (min)	0	3	5	10	15	30	0	3	5	10	15	30	
p-p44 (Fold of basal)	1.0	0.9 ±0.12	1.1 ±0.15	1.6 ±0.46	2.8 ±0.34	2.5 ±0.06	1.0	1.4 ±0.27	0.8 ±0.05	1.1 ±0.08	1.5 ±0.26	1.2 ±0.03	3
p-p42 (Fold of basal)	1.0	0.9 ±0.04	1.1 ±0.17	2.2 ±0. 4	2.8 ±0.34	2.5 ±0.27	1.0	1.2 ±0.19	1.1 ±0.28	1.4 ±0. 04	1.6 ±0.02	1.3 ±0.15	3

Figure 4D.

siRNA	—	Scrb	p42	—	Scrb	p42	N
TNF-α	—			15 ng/ml			
p42 (Fold of basal)	1.0	0.8±0.12	0.5±0.13	1.0±0.2	1.1±0.25	0.4±0.04	3
VCAM-1 (Fold of basal)	1.0	1.0±0.03	0.9 ±0.18	3.3±0.43	3.5 ±0.27	1.4 ±0.11	3

Figure 4E.

Treatment	Control						TNFR nAb						N
Time (min)	0	3	5	10	15	30	0	3	5	10	15	30	
p-p44 (Fold of basal)	1.0	1.0 ±0.1	1.3 ±0.2	2.2 ±0.44	1.3 ±0.34	1.4 ±0.3	1.0	1.0 ±0.12	1.1 ±0.15	1.3 ±0.22	1.0 ±0.06	0.5 ±0.18	3
p-p42 (Fold of basal	1.0	1.0 ±0.04	1.0 ±0.17	1.4 ±0.44	1.5 ±0.31	1.1 ±0.39	1.0	1.0 ±0.27	1.0 ±0.19	1.1 ±0. 34	1.0 ±0.3	0.6 ±0.08	3

Treatment	Gö6976						Edaravone						N
Time (min)	0	3	5	10	15	30	0	3	5	10	15	30	
p-p44 (Fold of basal)	1.0	1.0 ±0.11	0.9 ±0.03	0.9 ±0.19	1.0 ±0.04	0.9 ±0.2	1.0	1.0 ±0.17	1.1 ±0.06	1.1 ±0.16	1.4 ±0.22	1.3 ±0.13	3
p-p42 (Fold of basal	1.0	0.9 ±0.24	0.9 ±0.07	0.8 ±0. 3	0.9 ±0.31	0.5 ±0.29	1.0	1.4 ±0.1	1.3 ±0.3	1.9 ±0.08	2.4 ±0.39	1.7 ±0.05	3

Treatment	DPI					
Time (min)	0	3	5	10	15	30
p-p44 (Fold of basal)	1.0	1.1 ±0.12	1.0 ±0.15	1.3 ±0.43	1.2 ±0.36	1.1 ±0.19
p-p42 (Fold of basal	1.0	0.9 ±0.12	1.3 ±0.15	1.6 ±0. 27	1.6 ±0.53	1.4 ±0.38

Figure 5C.

Treatment	Control						SB202190						N
Time (min)	0	3	5	10	15	30	0	3	5	10	15	30	
p-p38 (Fold of basal)	1.0	2.1 ±0.17	4.6 ±0.14	5.3 ±0.16	2.5 ±0.34	2.5 ±0.05	1.0	1.0 ±0.07	1.3 ±0.02	1.3 ±0.06	2.4 ±0.01	2.5 ±0.15	3

Figure 5D.

siRNA	—	Scrb	p38	—	Scrb	p38	N
TNF-α	—			15 ng/ml			
p38 (Fold of basal)	1.0	0.9±0.14	0.2±0.19	0.9±0.1	0.7±0.13	0.2±0.02	3
VCAM-1 (Fold of basal)	1.0	1.1±0.21	0.9 ±0.12	2.8±0.17	2.8 ±0.07	1.4 ±0.09	3

Figure 5E.

Treatment	Control						TNFR nAb						N
Time (min)	0	3	5	10	15	30	0	3	5	10	15	30	
p-p44 (Fold of basal)	1.0	1.0 ±0.1	1.3 ±0.2	2.2 ±0.44	1.3 ±0.34	1.4 ±0.3	1.0	1.0 ±0.12	1.1 ±0.15	1.3 ±0.22	1.0 ±0.06	0.5 ±0.18	3

Treatment	Gö6976						Edaravone						N
Time (min)	0	3	5	10	15	30	0	3	5	10	15	30	
p-p44 (Fold of basal)	1.0	1.0 ±0.11	0.9 ±0.03	0.9 ±0.19	1.0 ±0.04	0.9 ±0.2	1.0	1.0 ±0.17	1.1 ±0.06	1.1 ±0.16	1.4 ±0.22	1.3 ±0.13	3

Treatment	DPI					
Time (min)	0	3	5	10	15	30
p-p44 (Fold of basal)	1.0	1.1 ±0.12	1.0 ±0.15	1.3 ±0.43	1.2 ±0.36	1.1 ±0.19
