# Supplementary material for: NADPH Oxidase/ROS-Dependent VCAM-1 Induction on TNF-α-Challenged Human Cardiac Fibroblasts Enhances Monocyte Adhesion
Source: Front Pharmacol. 2016 Jan 28;6:310. doi: 10.3389/fphar.2015.00310 (PMC4729888; doi:10.3389/fphar.2015.00310)
Supplement: Supplementary file 2 [file Table_2.PDF]

Supplementary Table 2

Figure 6C.

Treatment	Control						SP600125						N
Time (min)	0	3	5	10	15	30	0	3	5	10	15	30	
p-JNK2 (Fold of basal)	1.0	1.1 ±0.12	1.1 ±0.15	1.3 ±0.46	2.4 ±0.34	1.5 ±0.06	1.0	1.1 ±0.27	1.1 ±0.05	1.0 ±0.08	1.3 ±0.26	1.2 ±0.03	3
p-JNK1 (Fold of basal)	1.0	1.7 ±0.04	1.1 ±0.17	2.4 ±0. 4	1.5 ±0.34	2.5 ±0.27	1.0	1.3 ±0.19	1.2 ±0.28	1.1 ±0. 04	1.7 ±0.02	1.8 ±0.15	3

Figure 6D.

siRNA	—	Scrb	JNK1	—	Scrb	JNK1	N
TNF-α	—			15 ng/ml			
JNK1 (Fold of basal)	1.0	1.0±0.07	0.6±0.03	0.8±0.06	0.9±0.07	0.3±0.02	3
VCAM-1 (Fold of basal)	1.0	0.9±0.17	0.8 ±0.14	1.4±0.16	1.2 ±0.07	0.9 ±0.21	3

Figure 6E.

Treatment	Control						Gö6976						N
Time (min)	0	3	5	10	15	30	0	3	5	10	15	30	
p-JNK2 (Fold of basal)	1.0	0.9 ±0.06	1.0 ±0.13	2.1 ±0.14	2.3 ±0.13	1.8 ±0.12	1.0	1.0 ±0.07	0.9 ±0.06	1.4 ±0.15	1.7 ±0.07	1.2 ±0.05	3
p-JNK1 (Fold of basal)	1.0	0.9 ±0.12	0.9 ±0.09	1.5 ±0.1	2.0 ±0.39	1.2 ±0.42	1.0	1.0 ±0.07	0.9 ±0.02	1.2 ±0.06	1.5 ±0.01	1.2 ±0.2	3

Treatment	Gö6976						Edaravone						N
Time (min)	0	3	5	10	15	30	0	3	5	10	15	30	
p-JNK2 (Fold of basal)	1.0	0.9 ±0.18	0.9 ±0.12	1.1 ±0.19	0.8 ±0.08	1.2 ±0.03	1.0	1.1 ±0.16	1.3 ±0.1	2.0 ±0.12	2.2 ±0.13	1.4 ±0.21	3
p-JNK1 (Fold of basal)	1.0	0.9 ±0.06	0.9 ±0.14	1.0 ±0.12	0.9 ±0.08	1.0 ±0.04	1.0	1.0 ±0.1	1.1 ±0.15	2.0 ±0.38	2.4 ±0.3	1.5 ±0.28	3

Treatment	DPI					
Time (min)	0	3	5	10	15	30
p-JNK2 (Fold of basal)	1.0	1.1 ±0.11	1.2 ±0.1	1.3 ±0.2	1.5 ±0.24	1.1 ±0.09
p-JNK1 (Fold of basal)	1.0	1.1 ±0.22	1.1 ±0.02	1.2 ±0.08	1.4 ±0.07	1.0 ±0.03

Figure 7C.

Treatment	Control						TSIIA						N
Time (min)	0	3	5	10	15	30	0	3	5	10	15	30	
p-c-Jun (Fold of basal)	1.0	1.1 ±0.17	1.4 ±0.14	3.2 ±0.16	2.2 ±0.34	2.5 ±0.05	1.0	0.9 ±0.07	1.0 ±0.02	1.0 ±0.06	1.5 ±0.01	0.8 ±0.15	3

Figure 7D.

siRNA	—	Scrb	c-Jun	—	Scrb	c-Jun	N
TNF-α	—			15 ng/ml			
c-Jun (Fold of basal)	1.0	1.1±0.2	0.5±0.05	1.2±0.3	1.2±0.17	0.4±0.04	3
VCAM-1 (Fold of basal)	1.0	1.0±0.07	1.2 ±0.02	3.2±0.42	2.5 ±0.65	1.6 ±0.05	3

Figure 7E.

Treatment	Control						TNFR nAb						N
Time (min)	0	3	5	10	15	30	0	3	5	10	15	30	
p-c-Jun (Fold of basal)	1.0	1.2 ±0.27	1.3 ±0.18	2.6 ±0.49	2.6 ±0.7	2.9 ±0.7	1.0	1.0 ±0.05	0.9 ±0.02	0.9 ±0.08	1.1 ±0.12	1.1 ±0.1	3

Treatment	DPI						Edaravone						N
Time (min)	0	3	5	10	15	30	0	3	5	10	15	30	
p-c-Jun (Fold of basal)	1.0	0.8 ±0.18	0.9 ±0.05	0.9 ±0.13	0.9 ±0.02	0.7 ±0.08	1.0	0.8 ±0.17	0.8 ±0.13	1.1 ±0.16	1.1 ±0.04	0.8 ±0.03	3

Treatment	SP600125						si-p38α						N
Time (min)	0	3	5	10	15	30	0	3	5	10	15	30	
p-c-Jun (Fold of basal)	1.0	1.0 ±0.2	0.9 ±0.04	0.8 ±0.1	0.8 ±0.3	1.1 ±0.08	1.0	1.0 ±0.18	1.0 ±0.06	1.2 ±0.3	1.6 ±0.16	1.0 ±0.2	3

Treatment	Gö6976						U0126						N
Time (min)	0	3	5	10	15	30	0	3	5	10	15	30	
p-c-Jun (Fold of basal)	1.0	1.0 ±0.11	1.2 ±0.14	1.2 ±0.34	0.9 ±0.06	1.2 ±0.18	1.0	0.9 ±0.03	1.2 ±0.18	1.0 ±0.19	1.2 ±0.25	0.9 ±0.09	3
